# Moxifloxacin and ciprofloxacin induces S-phase arrest and augments apoptotic effects of cisplatin in human pancreatic cancer cells via ERK activation

**DOI:** 10.1186/s12885-015-1560-y

**Published:** 2015-08-11

**Authors:** Vikas Yadav, Pallavi Varshney, Sarwat Sultana, Jyoti Yadav, Neeru Saini

**Affiliations:** 1 CSIR-Institute of Genomics and Integrative Biology (CSIR-IGIB), Mall Road, Delhi, India; 2 Department of Medical Elementology and Toxicology, Jamia Hamdard (Hamdard University), Hamdard Nagar, New Delhi India

**Keywords:** Fluoroquinolone, Moxifloxacin, Ciprofloxacin, Apoptosis, Cell cycle arrest, Pancreatic cancer, ERK

## Abstract

**Background:**

Pancreatic cancer, one of the most dreadful gastrointestinal tract malignancies, with the current chemotherapeutic drugs has posed a major impediment owing to poor prognosis and chemo-resistance thereby suggesting critical need for additional drugs as therapeutics in combating the situation. Fluoroquinolones have shown promising and significant anti-tumor effects on several carcinoma cell lines.

**Methods:**

Previously, we reported growth inhibitory effects of fourth generation fluoroquinolone Gatifloxacin, while in the current study we have investigated the anti-proliferative and apoptosis-inducing mechanism of older generation fluoroquinolones Moxifloxacin and Ciprofloxacin on the pancreatic cancer cell-lines MIA PaCa-2 and Panc-1. Cytotoxicity was measured by MTT assay. Apoptosis induction was evaluated using annexin assay, cell cycle assay and activation of caspase-3, 8, 9 were measured by western blotting and enzyme activity assay.

**Results:**

Herein, we found that both the fluoroquinolones suppressed the proliferation of pancreatic cancer cells by causing S-phase arrest and apoptosis. Blockade in S-phase of cell cycle was associated with decrease in the levels of p27, p21, CDK2, cyclin-A and cyclin-E. Herein we also observed triggering of extrinsic as well as intrinsic mitochondrial apoptotic pathway as suggested by the activation of caspase-8, 9, 3, and Bid respectively. All this was accompanied by downregulation of antiapoptotic protein Bcl-xL and upregulation of proapoptotic protein Bak. Our results strongly suggest the role of extracellular-signal-regulated kinases (ERK1/2), but not p53, p38 and c-JUN N-terminal kinase (JNK) in fluoroquinolone induced growth inhibitory effects in both the cell lines. Additionally, we also found both the fluoroquinolones to augment the apoptotic effects of broad spectrum anticancer drug Cisplatin via ERK.

**Conclusion:**

The fact that these fluoroquinolones synergize the effect of cisplatin opens new insight into therapeutic index in treatment of pancreatic cancer.

**Electronic supplementary material:**

The online version of this article (doi:10.1186/s12885-015-1560-y) contains supplementary material, which is available to authorized users.

## Background

Pancreatic cancer is one of the most dreadful gastrointestinal tract malignancies, owing to its poor diagnosis, rare curative surgeries and less understood etiology [1]. The survival rate period of 5-years is less than 5 %, which is an issue of apprehension. Till date the only curative option is to undergo surgery, although resection rates are under 20 % and the median survival rate is rarely more than 20 months. Impact of the post-operative complications on long-term survival after resection of pancreatic cancer is not well reported. According to several studies, the postoperative mortality rates are less than 6 % in specialized centres with an overall morbidity rate of 20-50 % [2, 3]. Unresectable cases generally receive chemotherapeutic treatment comprising of a standard Gemcitabine (2′, 2′-difluorocytidine) alone or in combination with Erlotinib or Folfirinox [4]. Recently Goldstein et al., showed superior efficacy of combined therapy of Nab-paclitaxel (Abraxane) plus Gemcitabine over gemcitabine alone [5]. However to our dismay, almost all patients suffering from advanced stage pancreatic carcinoma develop an inherent resistance to Gemcitabine, the mechanisms of which is yet unknown [6]. As each of the therapies has limitations, hence there is always a need for new strategies to improve the treatment efficacy of this fatal disease.

Fluoroquinolones (FQ) are broad spectrum antibiotics and are active against various gram positive and gram negative bacteria, specifically by targeting bacterial DNA gyrase and topoisomerase [7, 8]. Apart, from their antibacterial, antimycobacterial and other clinical implications, traditional FQ family members MFX and CFX are also known to have several immunomodulatory effects *in vitro* in various cell lines [9–11]. Previous reports focusing on the ability of FQs to induce apoptosis and cell cycle arrest in various cancer cell lines alone or in combination with other chemotherapeutic agents have rendered them unique among other antibiotic family members [12–18].

Previously we reported that the newer generation FQ, Gatifloxacin possesses antiproliferative activity against pancreatic cancer cell lines by causing S/G2 phase cell cycle arrest without induction of apoptosis through p21, p27 and p53 dependent pathway [20]. Herein, we have investigated the effect of MFX and CFX on survival and proliferation of pancreatic cancer cell lines (MIA PaCa-2 and Panc-1) and found that both were able to suppress the proliferation of pancreatic cancer cells and induce apoptosis through similar mechanism. In addition our results also suggest that both the FQ augments the apoptotic effects of Cisplatin (CDDP) via ERK activation.

## Methods

### Reagents and antibodies

DMEM, Antibiotic Antimycotic solution, Trypsin EDTA, Dimethyl sulfoxide (DMSO), propidium iodide (PI), protease and phosphatase inhibitor cocktail, BCIP-NBT, BCA reagent, carbonyl cyanide m-chlorophenyl hydrazone (mClCCP; a mitochondrial uncoupler), 3,3′-dihexyloxacarbocyanine iodide (DiOC6), MTT, ERK inhibitor (U0126), p38 inhibitor (SB203580), Cisplatin (CDDP) were purchased from Sigma (St. Louis, Missouri, USA). Caspase-8 inhibitor and zVAD-fmk (carbobenzoxy-valyl-alanyl-aspartyl-[O-methyl]-fluoromethyl-ketone) were from calbiochem, Germany. Foetal bovine serum was purchased from Biological Industries (Kibbutz Beit Haemek, Israel). Antibodies Cyclin-A, Cyclin-E, CDK-2, Cyclin-B1, p21, p27, Bid, PARP, cleaved caspase-3, −8, −9 were purchased from Cell signaling technologies (MA, USA). Antibodies Bax, Bak, Bcl-xL, cMyc, GAPDH, pAKT (Ser 473), AKT, p53, pCDC2, CDC2, CDC25c, pP38, total P38, pJNK, total JNK, pERK1/2, total ERK were purchased from Santacruz biotechnology (Santa Cruz, CA, USA). MFX and CFX were obtained from Cipla (India).

### Cell culture

MIA PaCa-2 and Panc-1 cells were obtained from National Centre for Cell Science, Pune, India and maintained in DMEM medium containing 10 % (v/v) FBS, 100 units/ml penicillin, 100 μg/ml streptomycin, 0.25 μg/ml amphotericin-B in a humidified 5 % CO_2_ atmosphere. Both the cell lines harbour mutations in their p53 gene. In MIA PaCa-2 cells, Arginine is substituted with Tryptophan at 248-position and in Panc-1 cells, Arginine is substituted with Cysteine at 273-position [19]. Cells growing in logarithmic phase were used in all experiments. Synchronized and growth arrested cultures were then subjected to MFX and CFX (0–400 μg/ml) treatment in complete media for 24 h and 48 h respectively. Wherever indicated, flow cytometry and western blot analysis (described below) were done using U0126 (5 μM for MIA PaCa-2 and 10 μM for Panc-1) in DMSO. For control, equivalent volume of DMSO was added to the culture medium 1 h prior to the treatment.

### Cell viability assay

Cell viability assay was performed using MTT [3-(4, 5-dimethyl thiazol-2yl)-2, 5-diphenyltetrazolium bromide]. 10,000 cells per well were seeded in 96 well plates and treated with different concentrations (0–400 μg/ml) of MFX and CFX in triplicates. As controls, Dextrose 5 % (w/v) treated cells (Vehicle) were included in each experiments. Post treatment, 10 μL of MTT (5 μg/ml) was added to each well and incubated for 3 h at 37 °C in dark. Formazan crystals formed were dissolved in 100 μl DMSO and the absorbance was measured at 570 nM using an ELISA reader. Cell viability was calculated as reported earlier [21].

### Annexin assay

Apoptosis was assessed using Guava Nexin kit and Guava PCA system according to the manufacturer’s protocol (Guava Technologies, Hayward, California, USA). The assay uses AnnexinV-PE to detect the translocation of phosphatidylserine onto the surface of apoptotic cells. 7-amino actinomycin-D (7-AAD), the cell impermeable dye is included in the Guava Nexin Reagent, which is excluded from live healthy cells and early apoptotic cells but permeates late-stage apoptotic and dead cells.). AnnexinV-PE fluorescence was analyzed by cytosoft software (Guava Technologies, Hayward. California, USA). A minimum of 2000 events were counted.

### Cell cycle analysis

For analysis of cell cycle distribution, 1 × 10^6^ cells were harvested by centrifugation, washed with phosphate-buffer saline (PBS), fixed with ice cold 70 % ethanol and treated with 1 mg/ml RNAse for 30 min. Intracellular DNA was labelled with propidium iodide (50 μg/ml) and incubated at 4 °C in dark. Samples were then analyzed using flow cytometer (Guava Technologies, Hayward, California, USA) and cytosoft software (Guava Technologies, Hayward, California, USA). A minimum of 5,000 events were counted [20].

### DNA fragmentation and caspase activity assay

For DNA fragmentation analysis, 48 h post CFX/MFX treatment DNA was isolated according to manufacturer’s protocol (BioVision Incorporated, Milpitas, California, USA). In brief, FQ treated cells were harvested and resuspended in 50 μl of ice cold lysis buffer containing 10 mM Tris–HCl (pH 7.4), 150 mM NaCl, 5 mM EDTA and 0.5 % Triton X-100 by gentle pipetting. Isolated DNA was precipitated and analyzed electrophoretically on 1.8 % agarose gel containing ethidium bromide using UV-spectrophotometer.

Caspase-3, −8 and −9 activities were determined using the respective colorimetric substrates (Calbiochem, Germany). FQ treated cells were lysed using caspase lysis buffer (50 mM HEPES, pH 7.4; 100 mM Nacl; 0.1 % CHAPS; 1 mM DTT, 0.1 mM EDTA) supplemented with protease inhibitor cocktail. 100 μg of total protein was incubated with colorimetric caspase-3 substrate Ac-DEVD-pNA/caspase-8 substrate Ac-IETD-pNA/caspase-9 substrate Ac-LEHD-pNA in an assay buffer (50 mM HEPES, pH 7.4; 100 mM Nacl; 0.1 % CHAPS; 10 mM DTT; 0.1 mM EDTA; 10 % Glycerol), at 37 °C for 3 h in dark. Caspase activity assay is based on the ability of active enzyme to cleave the chromophore from the enzyme substrates Ac-DEVD-pNA, Ac-IETD-pNA, Ac-LEHD-pNA respectively. pNA released upon caspase cleavage produces a yellow color, which is measured by spectrophotometer at 405 nM. The amount of yellow color produced is proportional to the amount of caspase activity present in the sample. One unit is defined as the amount of enzyme that will cleave 1picomole of the substrate per minute at 37 °C and pH 7.4. Results are presented as the fold change of the activity, in comparison with the untreated control [22].

### Mitochondrial membrane potential (Δψ_m_)

The mitochondrial membrane potential was measured with DiOC6 (3, 3′-dihexyloxacarbocyanine iodide; Sigma), a fluorochrome that is incorporated into the cells depending upon the Δψ_m_. Loss of DiOC6 fluorescence indicates reduction in the mitochondrial inner transmembrane potential, which was monitored using flow cytometer as described before. In brief, FQ treated MIA PaCa-2 and Panc-1 cells were stained with DiOC6 at a final concentration of 40 nM for 30 min at 37 °C in dark. Cells were washed, and the fluorescence intensity was analysed by a flow cytometer (Guava Technologies). A minimum of 5000 events were counted.

### Preparation of cell lysates and immunoblot analysis

Cell pellets obtained 48 h post treatment with FQ (0–400 μg/ml) were lysed with cell lytic buffer containing protease/phosphatase inhibitor cocktail purchased from Sigma (St. Louis, Missouri, USA). Protein concentration was determined using BCA (Sigma, St. Louis, Missouri, USA) protein estimation kit. Equal amount of sample lysate (90 μg for p21, p27 and 50 μg for rest of the proteins) were separated by SDS-PAGE and transferred to PVDF membrane. The membrane was blocked with 5 % skim milk (3 % BSA in case of phospho form of protein) in TBST and probed with primary antibody overnight followed by incubation with appropriate secondary antibody (ALP or HRP linked). After washing, blots were developed using enzyme based chemiluminescence assays (alkaline phosphatase) by BCIP-NBT (Sigma, Missouri, USA) or enhanced chemiluminescence ECL western blot detection system (Pierce, Illinois, USA). Measurement of signal intensity of protein expression on PVDF membrane was done using alphaimager 3400 (Alpha Innotech Corporation, San Leandro, California, USA) and normalized using GAPDH as loading control. All data were expressed as fold change. All the experiments were repeated three times; representative results are presented [23].

### Statistical analysis

Results are given as mean of three independent experiments ± SEM. Statistical analysis was performed with student’s two tailed *t*-test using SPSS (windows version 7.5); values of p ≤ 0.05 were considered statistically significant.

## Results

### Fluoroquinolones inhibits proliferation of human pancreatic cancer cells

**Fig. 1:**
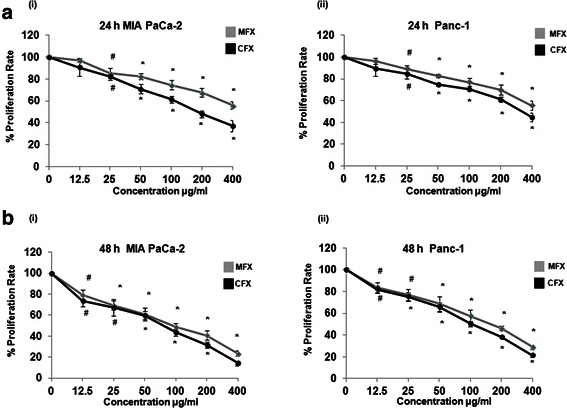
Antiproliferative effects of MFX and CFX on cultured pancreatic cancer cells. Dose and time dependent response of MFX and CFX on MIA PaCa-2 *(i)*, and Panc-1 *(ii)* cells, as assessed by MTT assay. Cells were seeded in 96 well plates (1 × 10^4^ cells/well) which were allowed to adhere overnight and were subsequently treated with increasing concentration of MFX and CFX for 24 h (**a**) and 48 h (**b**). Vertical axis represents % proliferation rate whereas Horizontal axis represents increasing concentration of MFX and CFX in μg/ml. Data are mean ± SEM three independent experiments performed in triplicate. *p < 0.01, #p < 0.05 versus control

To evaluate the effect of MFX and CFX on the proliferation of human pancreatic cancer cells MTT assay was performed. As shown in Fig. 1, both the FQ inhibited proliferation of MIA PaCa-2 and Panc-1 cells in a dose (0–400 μg/ml) and time (0–48 h) dependent manner. CFX was found to be more effective than MFX in suppressing cellular proliferation at higher doses (100, 200, 400 μg/ml, p < 0.01). Since these doses were in accordance with several previous reports [14, 15, 24–27] further experiments were carried out at these doses.

### Fluoroquinolones induce S-phase arrest and apoptosis in pancreatic carcinoma cells

Next, to investigate whether FQ-induced cell death was due to apoptosis, annexin assay was performed. As shown in Table 1, CFX treatment led to statistical significant increase in apoptosis at 200 μg/ml (p = 0.009) and 400 μg/ml (p < 0.01) whereas MFX treatment led to increase in percentage of apoptosis only at 400 μg/ml (p < 0.006) in both the cell lines and at 24 h and 48 h respectively. We did not find apoptosis at lower doses of CFX (100 μg/ml) and MFX (100 and 200 μg/ml) in both the cell lines. Results of annexin-V were also validated using curcumin as a positive control (data not shown).

**Table 1:** Results representing the annexin assay after treatment of pancreatic cancer cells with MFX/CFX

MIA Pa Ca-2	24 h	48 h	Panc-1	24 h	48 h
0 μg/ml	5 ± 2 %	1.6 ± 0.5 %	0 μg/ml	5.2 ± 0.58 %	4.2 ± 2.7 %
MFX 100 μg/ml	4.3 ± 0.64 %	4.4 ± 0.85 %	MFX 100 μg/ml	2.1 ± 2.7 %	4.6 ± 3.5 %
MFX 200 μg/ml	4.9 ± 0.6 %	5.9 ± 0.4 %	MFX 200 μg/ml	3.3 ± 1.59 %	7.9 ± 1.2 %
MFX 400 μg/ml	12.8 ± 1.2 %	23.4 ± 2 %	MFX 400 μg/ml	13 ± 1.15 %	16.9 ± 1.99 %
CFX 100 μg/ml	7.5 ± 0.3 %	7.9 ± 2.45 %	CFX 100 μg/ml	9.2 ± 1.8 %	9.8 ± 1.5 %
CFX 200 μg/ml	13.8 ± 0.6 %	22.5 ± 2 %	CFX 200 μg/ml	19 ± 3.4 %	14.6 ± 0.78 %
CFX 400 μg/ml	18.2 ± 0.2 %	40.6 ± 2.2 %	CFX 400 μg/ml	20.5 ± 1.8 %	21.6 ± 1.4 %

Values represent the percentage of apoptosis

As induction of apoptosis is often preceded by changes in cell cycle kinetics, we next investigated the cell cycle changes in presence of CFX/MFX in both the cell lines. In congruence to our annexin results we found significant increase in SubG1 peak either with MFX (400 μg/ml) or CFX (200 and 400 μg/ml) treatment in both the cell lines (Table 2 and 3). Interestingly in both the cell lines we observed S-phase arrest at the lower doses of MFX and CFX (100, 200 μg/ml) at 24 h and 48 h respectively.

**Table 2:** Results representing the Cell cycle analysis of MFX and CFX treated MIA PaCa-2 cells

24 h	Sub G1	G1	S	G2	48 h	Sub G1	G1	S	G2
MIA PaCa-2					MIA PaCa-2				
0 μg/ml	5 ± 0.5	53.8 ± 3.2	7.5 ± 1	33.7 ± 2.1	0 μg/ml	2.6 ± 0.5	67.1 ± 3	6.3 ± 1.2	24 ± 1.5
MFX 100 μg/ml	5.7 ± 0.35	48.2 ± 2.1	**10.4** ± 1.1	35.7 ± 3.1	MFX 100 μg/ml	2.1 ± 1.1	63.7 ± 2.5	**10.6** ± 0.9	23.6 ± 1
MFX 200 μg/ml	6.2 ± 0.4	60.6 ± 4	**11** ± 1.2	22.2 ± 2.3	MFX 200 μg/ml	3.5 ± 2	54.3 ± 2	**18.1** ± 0.8	24.1 ± 0.5
MFX 400 μg/ml	**28 ± 1.5**	49.1 ± 2.6	7.1 ± 1.5	15.8 ± 1.8	MFX 400 μg/ml	**37.6 ± 2.1**	40 ± 3.4	**11** ± 1.2	11.4 ± 1.8
CFX 100 μg/ml	4.5 ± 0.6	63 ± 1.5	**8.9** ± 2	23.6 ± 1.8	CFX 100 μg/ml	5.5 ± 1.7	51.5 ± 1.5	**14.3** ± 0.6	28.7 ± 3
CFX 200 μg/ml	**18.5 ± 2**	55.2 ± 2.1	**9.1** ± 1.3	17.2 ± 2.3	CFX 200 μg/ml	**28.4 ± 1.9**	52.8 ± 2	**14** ± 1.1	4.8 ± 4.5
CFX 400 μg/ml	**30.1 ± 2**	48.1 ± 3	7.3 ± 2	14.5 ± 2.7	CFX 400 μg/ml	**59.9 ± 1.1**	32.2 ± 3.9	4.4 ± 2	3.5 ± 3.2

Values represent the percent of population in each phase. Values with significant changes have been highlighted with bold format

**Table 3:** Results representing the Cell cycle analysis of MFX and CFX treated Panc-1 cells

24 h	Sub G1	G1	S	G2	48 h	Sub G1	G1	S	G2
Panc-1					Panc-1				
0 μg/ml	4.8 ± 1.5	61.6 ± 0.5	7.8 ± 0.7	25.8 ± 0.9	0 μg/ml	4.1 ± 0.8	66.2 ± 1	7.3 ± 0.5	22.4 ± 1.5
MFX 100 μg/ml	4.4 ± 1	59.7 ± 2	**9.7** ± 1	26.2 ± 2	MFX 100 μg/ml	4 ± 0.5	56.7 ± 2.4	**10.8** ± 1.5	28.5 ± 1
MFX 200 μg/ml	5.6 ± 1.2	60.2 ± 1.2	**11.6** ± 1.3	22.6 ± 1.4	MFX 200 μg/ml	4.1 ± 1	50.3 ± 3.1	**20.6** ± 2	24.6 ± 0.8
MFX 400 μg/ml	**10.4 ± 1 %**	57.9 ± 2.5	7.1 ± 0.6	24.6 ± 1.5	MFX 400 μg/ml	**20.5 ± 2.5**	52.8 ± 1.9	**12.4** ± 1	14.3 ± 2.2
CFX 100 μg/ml	5.1 ± 0.8	61 ± 1.3	**8.4** ± 1	25.5 ± 0.5	CFX 100 μg/ml	4.2 ± 1.1	53.6 ± 1.2	**13.4** ± 1.5	28.8 ± 1.7
CFX 200 μg/ml	**24 ± 1.2**	51 ± 2.1	**9** ± 0.5	16 ± 1.6	CFX 200 μg/ml	**17.7 ± 2**	50.2 ± 2.4	**10.6** ± 1.1	21.5 ± 0.9
CFX 400 μg/ml	**32 ± 1.5**	48.2 ± 3.2	7.3 ± 1	12.5 ± 2	CFX 400 μg/ml	**54.4 ± 1.5**	28.9 ± 3.3	8.1 ± 0.8	8.6 ± 2.6

Values represent the percent of population in each phase. Values with significant changes have been highlighted with bold format

### Fluoroquinolones activates intrinsic and extrinsic pathways of apoptosis

**Fig. 2:**
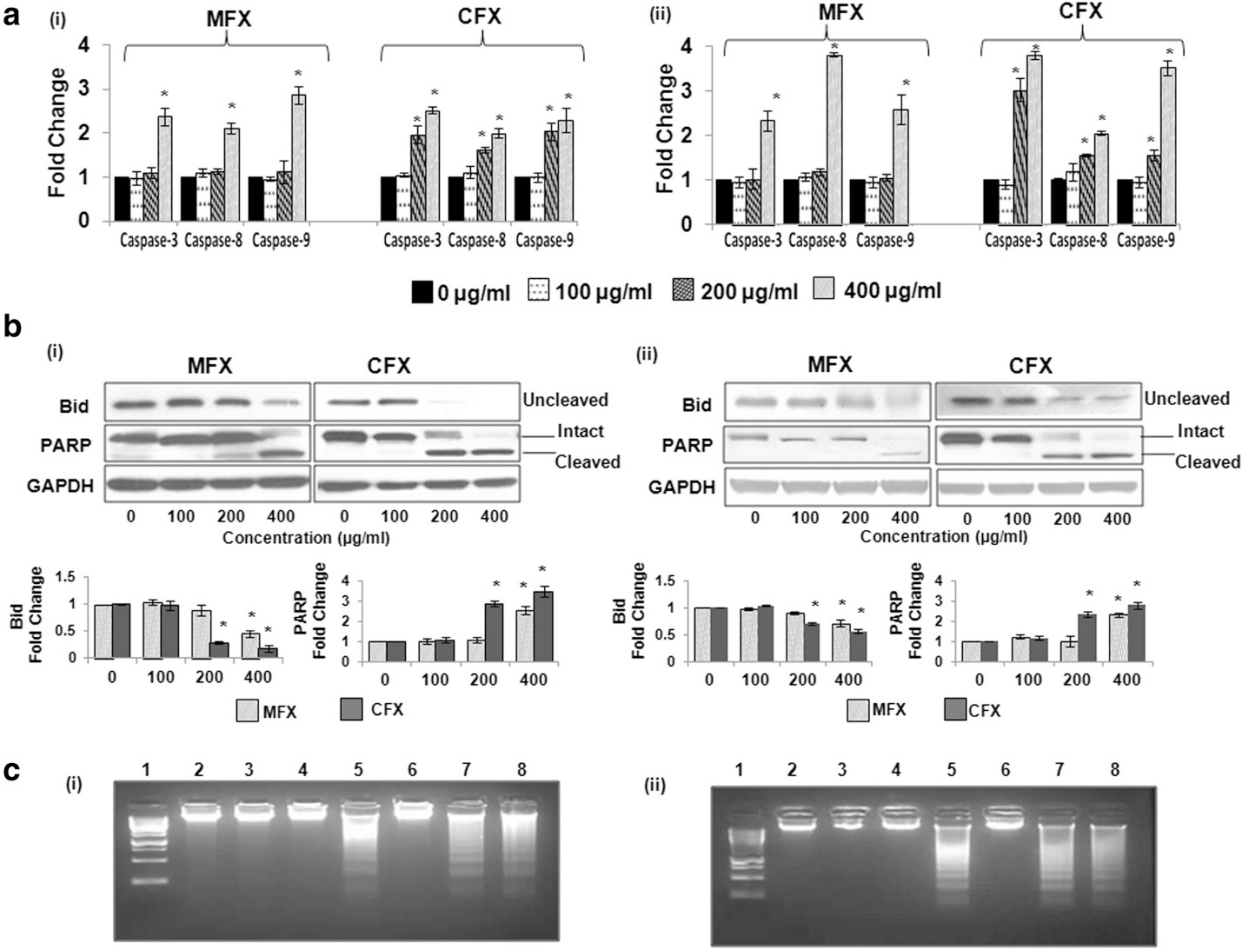
Effects of MFX and CFX on biochemical events associated with apoptosis. **a** As described in material and method, caspase-8, 9, 3 activities were measured in MIA PaCa-2 *(i)*, and Panc-1 cells *(ii)*, in presence and absence of MFX/CFX for 48 h. The enzyme activity was measured by extent of cleavage of the caspase substrates Ac-IETD-pNA, Ac-LEHD-pNA and Ac-DEVD-pNA respectively. Bar graph represents the mean ± SEM of the fold increase in enzyme activity versus untreated control of three independent experiments performed in duplicates. Here vertical axis represents fold change in caspase activity. *p < 0.015, #p < 0.05 **b** Western blot analysis of Bid activation and PARP cleavage in MIA PaCa-2 *(i)*, and Panc-1 cells *(ii)*, treated with MFX/CFX in a dose dependent manner for 48 h. GAPDH was used as loading control. Data are representative of typical experiment repeated three times with similar results. Bar Graph represents the mean ± SEM. here vertical axis represents fold change and horizontal axis represents concentration in μg/ml. *p < 0.01 versus control. **c** DNA was isolated from MFX/CFX treated MIA PaCa-2 *(i)*, and Panc-1 cells *(ii)* for 48 h, as described in material and method section, and was resolved onto 1.8 % agarose gel to detect DNA fragmentation, the characteristic feature of cells undergoing apoptosis. Pictures are representative of three independent experiments. (1) represents standard DNA marker, (2) DNA from untreated cells, (3) cells treated with 100 μg/ml of MFX, (4) cells treated with 200 μg/ml of MFX, (5) cells treated with 400 μg/ml of MFX, (6) cells treated with 100 μg/ml of CFX, (7) cells treated with 200 μg/ml of CFX, (8) cells treated with 400 μg/ml of CFX

Caspases are important players in the apoptotic pathway [28]. To address the involvement of caspases in FQ-induced apoptosis, activity of caspase-3, −8 and −9 were examined by colorimetric assay. As shown in Fig. 2a, significant increase in the activity of caspase-8 (p = 0.003), caspase-9 (p = 0.003), caspase-3 (p = 0.006) were observed in both the cell lines following MFX (400 μg/ml) and CFX (200 and 400 μg/ml) treatment for 48 h.

Several reports have demonstrated that caspase-8, and its substrate BID (a pro-apoptotic Bcl-2 protein containing only the BH3 domain), are frequently activated in response to certain apoptotic stimuli in a death receptor-independent manner. Once cleaved and activated it translocates to the mitochondria and leads to mitochondrial dysfunction and activation of caspase-9, which then transduces apoptotic signals further [29]. To investigate the possible involvement of Bid in FQ-induced cell death we next checked the levels of uncleaved Bid in presence and absence of both the FQs for 48 h. As expected, MFX (p < 0.008) and CFX (p < 0.01) treatment caused significant decrease in the levels of uncleaved BID in both the cell lines in a dose dependent manner (Fig. 2b).

Literature reveals that a number of cellular proteins, such as PARP, are cleaved following the activation of caspases and capase-3 activation has been shown to be required for DNA fragmentation [30]. Hence, we next checked the cleavage of PARP by western blot analysis and DNA fragmentation by agarose gel electrophoresis 48 h post CFX/MFX treatment. As shown in Fig. 2b, a statistically significant increase in cleaved PARP was seen in both the cell lines (p < 0.01). Furthermore, as expected, characteristic “ladder” pattern of apoptosis was also observed in both the cell lines treated with either MFX (400 μg/ml) or CFX (200-400 μg/ml) Fig. 2c.

Taken together our results indicate that a crosstalk exists between extrinsic and intrinsic pathway during MFX and CFX induced apoptosis via Bid.

### Fluoroquinolones induced apoptosis is caspase-8 dependent

**Fig. 3:**
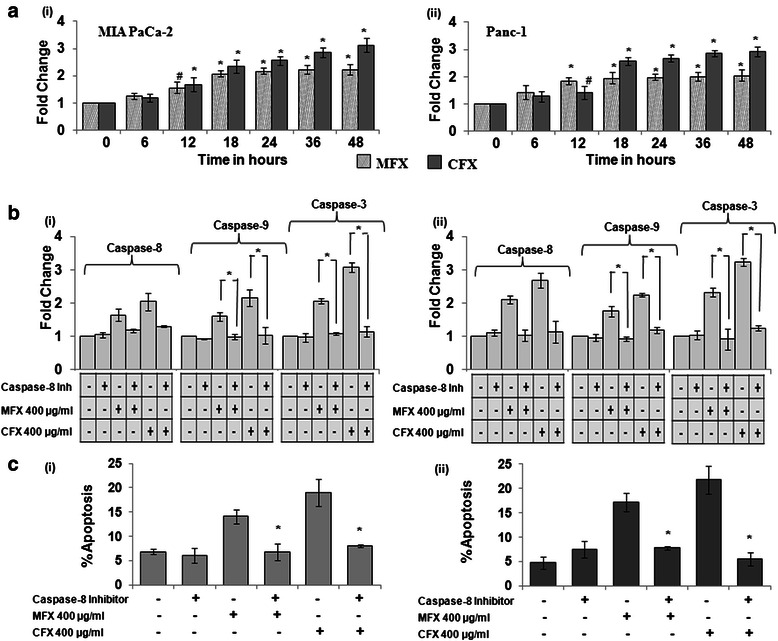
MFX and CFX induced apoptosis is caspase-8 dependent in both the cell lines. **a** MFX and CFX induced Caspase-8 activity in a time dependent manner in MIA Paca-2 *(i)*, and Panc-1 cells *(ii)*. Here vertical axis represents fold change in caspase activity and horizontal axis represents time in hours. *p < 0.015, #p < 0.05 **b** Caspase-8, 9, 3 activity under the effect of MFX and CFX in presence or absence of caspase-8 inhibitor in MIA PaCa-2 *(i)*, and Panc-1 *(ii)* cells. *p < 0.015, #p < 0.05 versus MFX/CFX. **c** Abolishment of apoptosis in MIA PaCa-2 *(i)*, and Panc-1 *(ii)*, cells in presence of caspase-8 inhibitor as assessed by annexin-V assay. Cell death is represented in form of bar graph where vertical axis represents % apoptotic cells and horizontal axis represents presence or absence of caspase-8 inhibitor (μM) along with MFX and CFX concentration in μg/ml. Bar graph represents mean ± SEM from three independent experiments. *p < 0.015, #p < 0.05 versus MFX/CFX

In order to confirm the role of caspase-8 in FQ induced apoptosis we first checked caspase-8 activity in a time dependent manner. As shown in Fig. 3a, MFX and CFX treatment led to significant increase in the caspase-8 activity from 6 h till 18 h (p < 0.01) in both the cell lines. Our experimental findings (Fig. 3b and c) further reveal that pre-treatment with caspase-8 inhibitor not only inhibited activation of caspase-8 but also inhibited caspase-9 and caspase-3 and simultaneously also rescued both the cell lines from FQ-induced apoptosis.

In order to strengthen the involvement of caspases in FQ induced apoptosis, we next checked the levels of PARP, cleaved caspase-8, −9, and −3 in presence or absence of zVAD-fmk along with MFX/CFX. As shown in Additional file 1: Figure S1, pre-treatment with zVAD-fmk inhibited activation of cleaved caspase-8, −9, −3 and PARP induced by MFX and CFX in both the cell lines. Taken together our results suggest that FQs induces apoptosis in a caspase-dependent manner.

### Fluoroquinolones disrupts mitochondrial membrane potential (Δψ_m_)

**Fig. 4:**
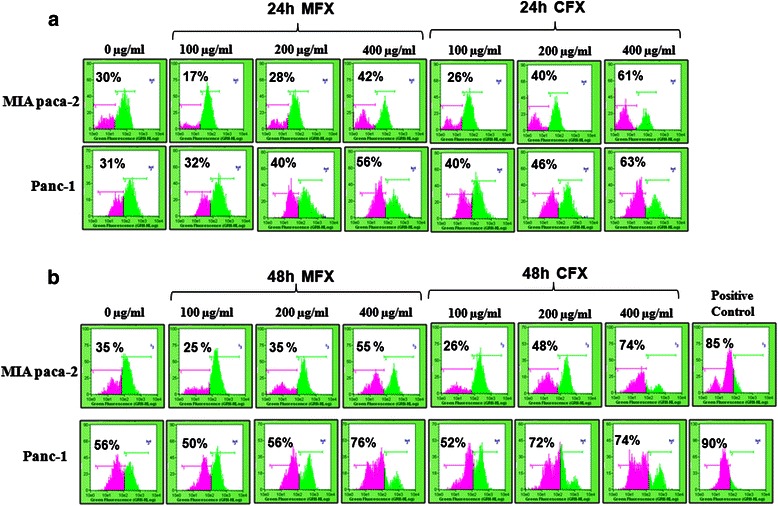
MFX and CFX perturb mitochondrial membrane potential. Mitochondrial membrane potential disruption was estimated using DiOC_6_**.** 20 min prior to harvesting, cells were incubated with 40 nM DiOC_6_ and after incubation MIA PaCa-2 and Panc-1 cells were harvested, and the change in fluorescence was measured by flowcytometry. The X-axis represents green fluorescence, and the Y-axis represents the count scale. The illustrated histograms are representative of the three independent experiments with similar results. Results were also validated using mCCCp as a positive control in both the cell lines

A variety of key events during apoptosis involve the mitochondria. Hence, to confirm the mitochondrial involvement in MFX and CFX mediated apoptotic cell death, we checked mitochondrial membrane integrity using the fluorescent probe DiOC6. The decrease in the green fluorescence is a marker of mitochondrial membrane potential dissipation and is measured as percentage of cells shifting towards the left. As shown in Fig. 4, while MFX treatment at 400 μg/ml showed a marked shift towards the left as compared to vehicle treated cells in both the cell lines, we did not find similar shift when cells were treated with 100, 200 μg/ml respectively. Similar to the above results, both the cell lines treated with CFX at 200 μg/ml and 400 μg/ml showed significant shift towards left.

Taken together, all these results indicate that MFX and CFX induce significant disruption of mitochondrial membrane potential in both the cell lines. mCCCP was used as positive control for DiOC6 experiments.

### Fluoroquinolones modulates expression of apoptotic and survival pathway proteins

**Fig. 5:**
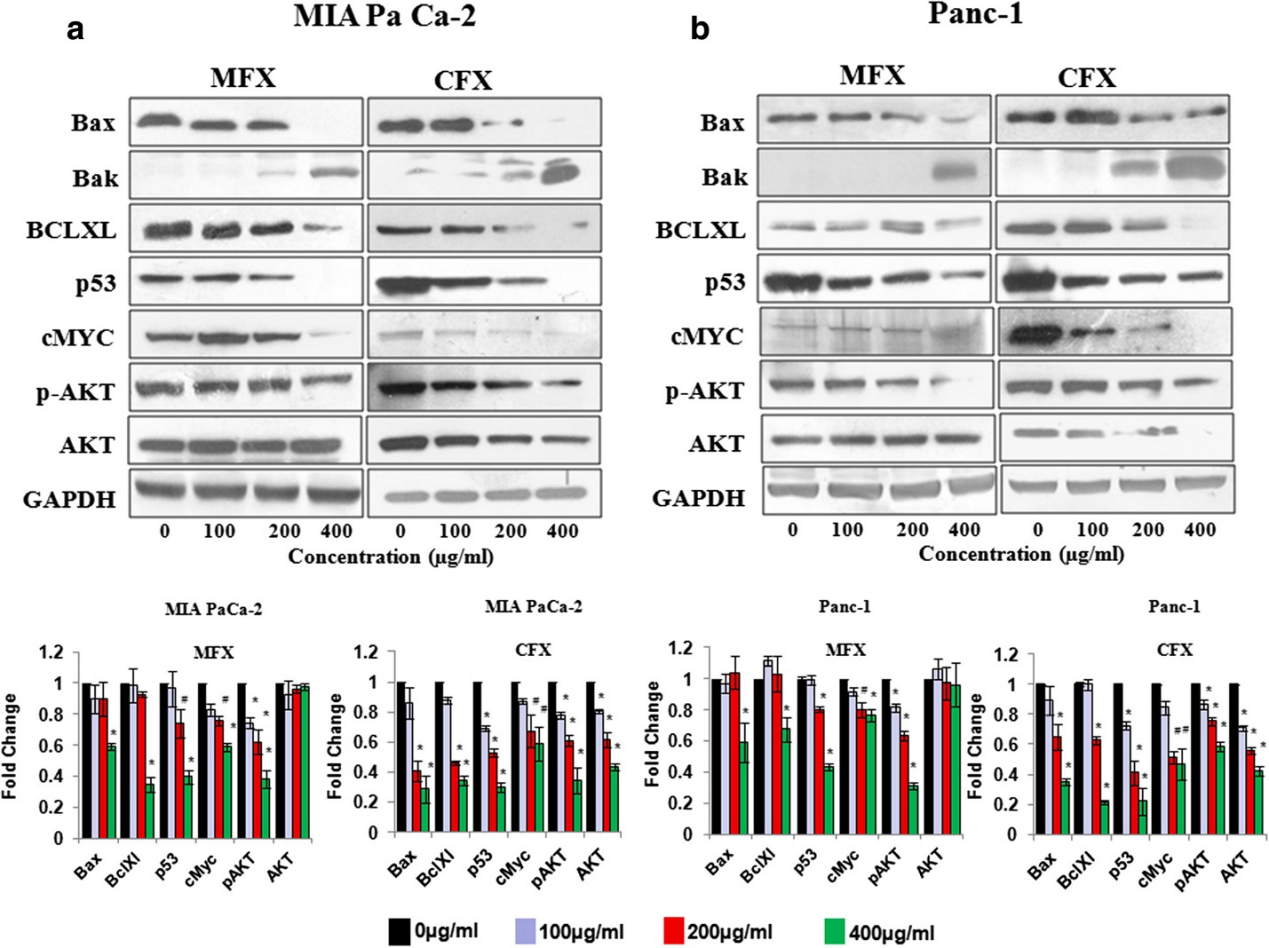
Effect of MFX and CFX on apoptotic and survival pathway proteins. Western blot analysis of apoptotic and survival pathway protein in MIA PaCa-2 (**a**), and panc-1 cells (**b**), treated with MFX and CFX in a dose dependent manner. GAPDH was used as loading control. The protein bands were quantified and normalized to GAPDH intensities. Data are representative of typical experiment repeated three times with similar results. Bar Graph represents the mean ± SEM of the fold change from three independent experiments. *p < 0.01, #p < 0.05 versus control

In order to better understand the molecular basis of FQ- induced apoptosis, the expression of several apoptotic and survival related proteins were checked by western blotting. As shown in Fig. 5, MFX and CFX treatment (400 μg/ml) led to statistically significant decrease in Bax (p < 0.01) and Bcl-xL (p < 0.018) proteins in both cell lines in a dose dependent manner. Previous studies, including our lab have shown that Bax and Bak are functionally redundant molecules and can substitute each other [31, 32]. Since in our study we found decrease in Bax, we also checked the levels of Bak after CFX and MFX treatment where we observed statistically significant increase in the levels of Bak (p < 0.012) in both the cell lines.

Literature reveals that tumor suppressor protein p53 not only act as a master regulator of cell cycle arrest and apoptosis in various stress stimuli but also act as transcription factor both for Bax and Bak [33]. Hence we also checked the levels of p53 in both the cell lines under the effect of FQ in a dose dependent manner. We found statistically significant decrease in the levels of p53 at 400 μg/ml of MFX (p < 0.001)/CFX (p < 0.006) treatment in both the cell lines (Fig. 5). To rule out the involvement of p53 in FQ-induced apoptosis we simultaneously performed annexin assay in HCT116 (human colon cancer cell line) wild type p53+/+ and deficient p53−/− cell lines in the presence of CFX/MFX. We treated both the cell lines with MFX and CFX in a dose dependent manner for 24 h and found insignificant changes in apoptotic cell population in any of the HCT116 cell lines. Simultaneously we also checked the expression of p53 protein and found that both MFX and CFX decreased the levels of p53 similar to that in pancreatic cancer cell lines (Additional file 2: Figure S2). Taken together our findings suggest that FQs induce apoptosis in a p53 independent manner.

In addition to all these we also observed that MFX and CFX down regulated the levels of proteins of the survival pathways (c-Myc and AKT-ser 473) in a dose dependent manner in both the cell lines. Although we did not find any significant change in the levels of total AKT after MFX treatment, but we observed CFX treatment down-regulated the levels of total AKT in a dose dependent manner in both the cell lines. These results suggest that FQs induce apoptosis by modulating apoptosis and cell survival pathway related proteins.

### Fluoroquinolones decreases the levels of S-Phase regulatory CDKs and cyclins in both the cell lines

**Fig. 6:**
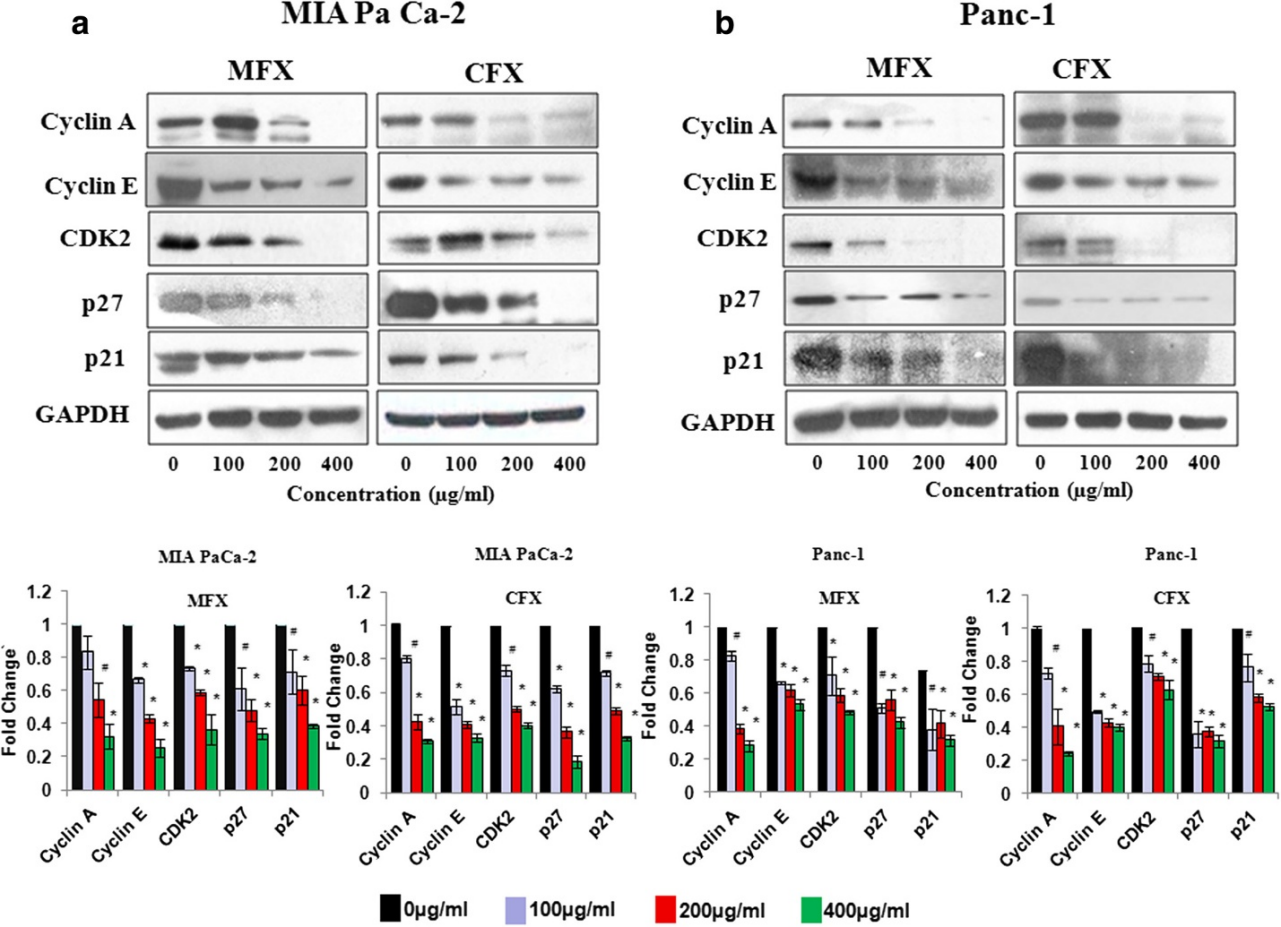
MFX and CFX effects S-phase associated regulatory proteins. Western blot analysis of S-phase regulatory Cyclins and CDKs in MIA PaCa-2 (**a**), and Panc-1 cells (**b**), treated with MFX and CFX in a dose dependent manner. GAPDH was used as loading control. The protein bands were quantified and normalized to GAPDH intensities. Data are representative of typical experiment repeated three times with similar results. Bar Graph represents the mean ± SEM of the fold change from three independent experiments. *p < 0.01, #p < 0.05 versus control

To identify the molecular mechanisms that govern the FQ-induced S-phase arrest, we next assessed the effect of FQs on the expression of cell cycle regulators of S-phase progression [34]. We also checked the levels of Cip/Kip family p21(Cip1) and p27(Kip1), which can inhibit cyclin E- and cyclin A-CDK activities. We found that treatment with MFX and CFX had a marked dose-dependent inhibitory effect on the protein expression of cyclin-A, cyclin-E, CDK2, p21 and p27 (Fig. 6) respectively. Although MFX and CFX treatment (200 and 400 μg/ml) resulted in significant decrease in the G2 phase population, they did not cause significant change in the levels of G2-phase proteins, i.e. CDC25c, cyclin-B1, pCDC2 (Additional file 3: Figure S3). Our findings further strengthen that FQ induce S-phase arrest by modulating the expression of S-phase cell cycle regulatory proteins in both the cell lines.

### Fluoroquinolones antiproliferative effects are ERK 1/2 dependent

**Fig. 7:**
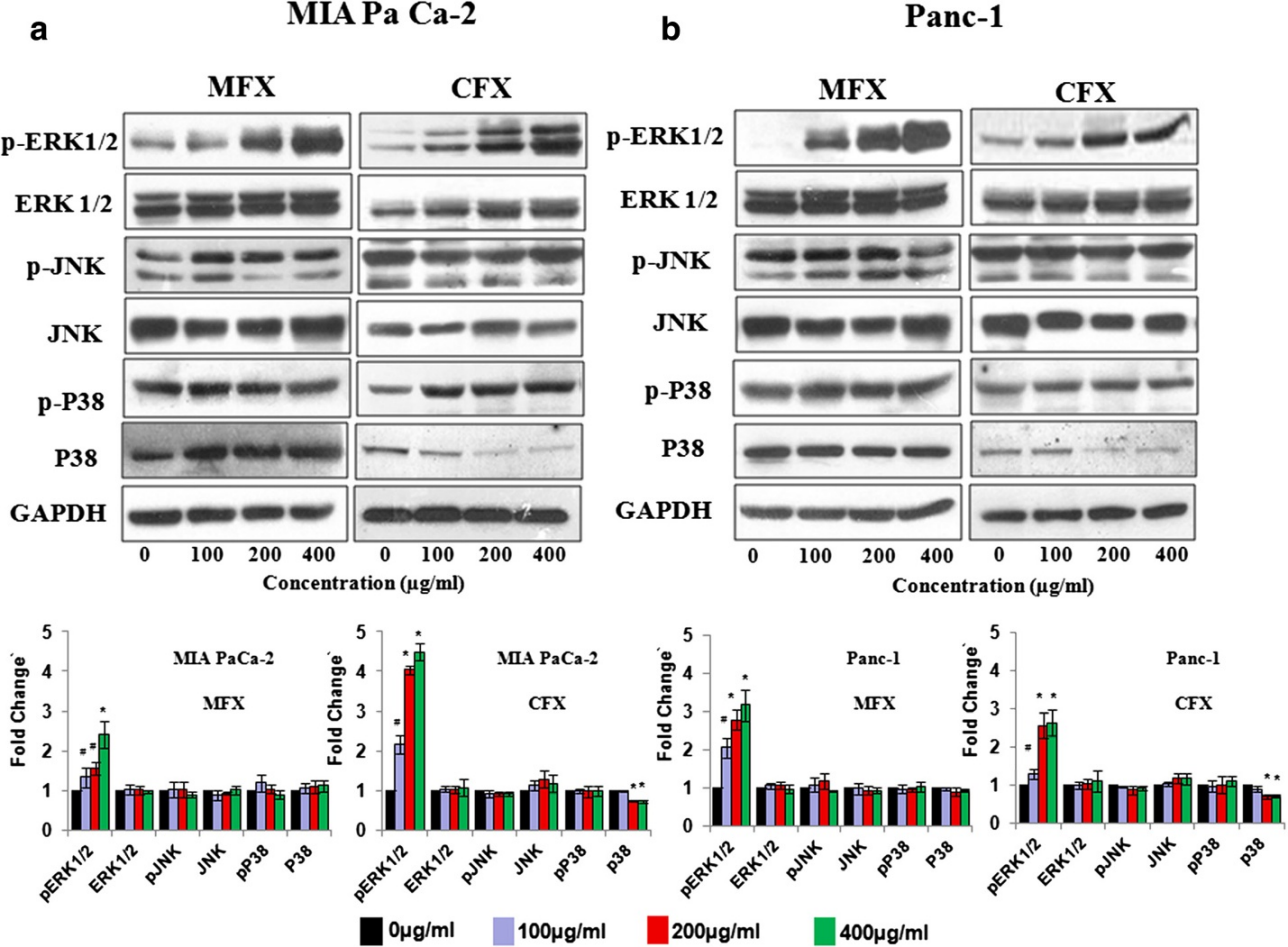
Effects of MFX and CFX on MAPK signalling pathway proteins Western blot analysis of MAPK pathway protein in MIA PaCa-2 (**a**), and panc-1 cells (**b**), treated with MFX and CFX in a dose dependent manner. GAPDH was used as loading control. The protein bands were quantified and normalized to GAPDH intensities. Data are representative of typical experiment repeated three times with similar results. Bar Graph represents the mean ± SEM of the fold change from three independent experiments. *p < 0.01, #p < 0.05 versus control

Literature reveals that three subfamilies of MAPKs: ERK1/2, JNK1/2, p38-MAPKs proteins cross-talks with other regulatory proteins to cause cell cycle arrest and apoptosis [35]. Hence, we next investigated the effect of both the FQs on MAPK signalling pathway proteins. As shown in Fig. 7, MFX (p < 0.05) and CFX (p < 0.01) treatment increased the expression of pERK1/2 in a dose dependent manner in both the cell lines without affecting the levels of total ERK. Also, there were insignificant changes in the levels of p-JNK, JNK, p-P38, p38 after MFX treatment in both the cell line. However CFX treatment decreased the expression of total-p38 protein.

**Fig. 8:**
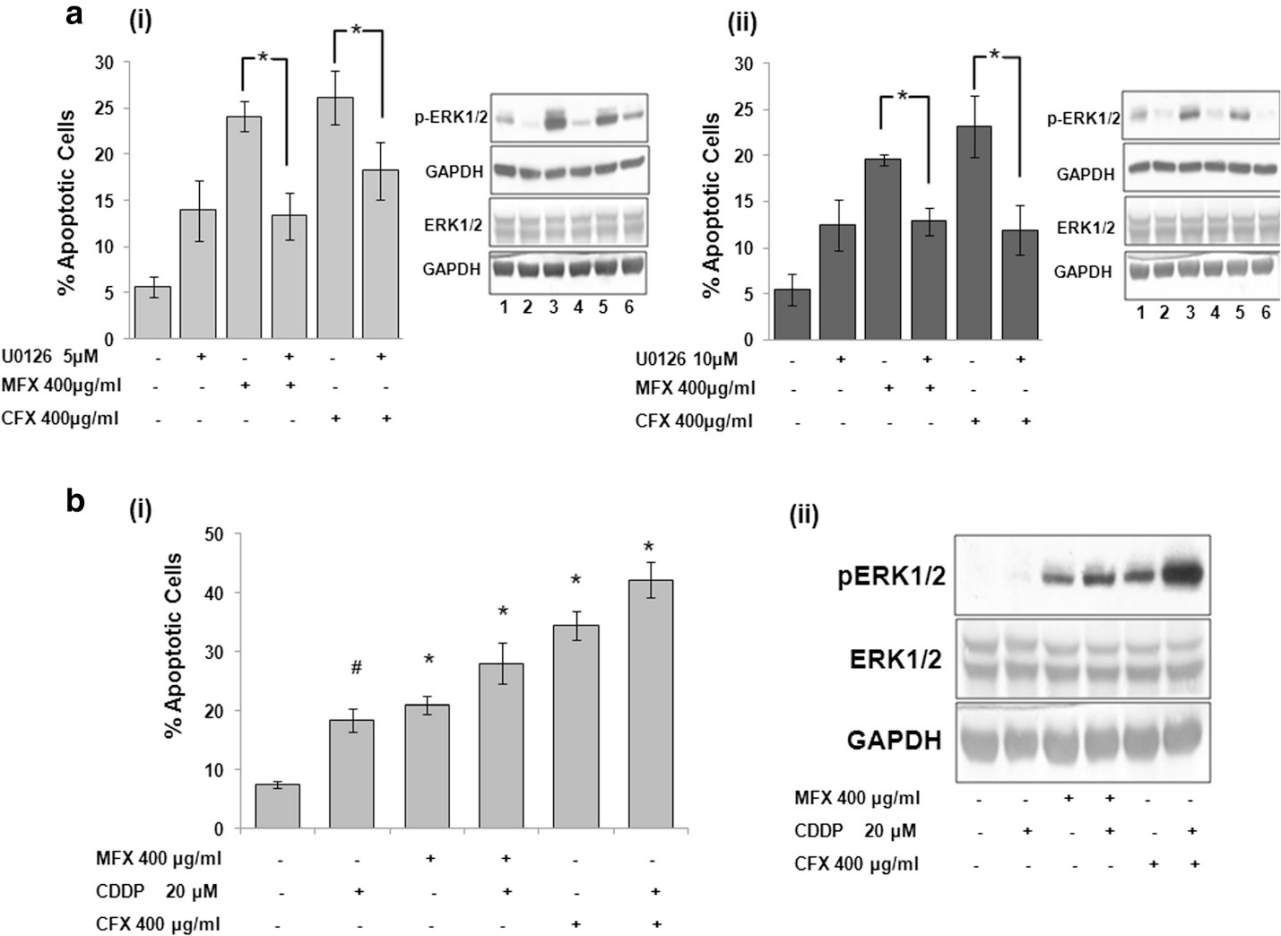
**a** MFX and CFX causes ERK mediated apoptosis in pancreatic cancer cells. *(i)* Abolishment of apoptosis in MIA PaCa-2 cells as assessed by annexin-V assay. Left panel represents the bar graph where vertical axis represents % apoptotic cells and horizontal axis represents MFX and CFX concentration in μg/ml, and U0126 concentration in μM. Bar graph represents mean ± SEM from three independent experiments. *p < 0.01 versus MFX/CFX alone. Right panel shows western blot for the knockdown efficiency of ERK1/2 inhibitor (U0126). *(ii)* Abolishment of apoptosis in Panc-1 cells as assessed by annexin-V assay. Left panel represents the bar graph where vertical axis represents % apoptotic cells and horizontal axis represents MFX and CFX concentration in μg/ml, and U0126 concentration in μM. Bar graph represents mean ± SEM from three independent experiments. *p < 0.01 versus MFX/CFX alone. Right panel shows western blot for the knockdown efficiency of ERK1/2 inhibitor (U0126). (1) represents untreated control cells, (2) U0126 treated cells, (3) cells treated with 400 μg/ml of MFX alone, (4) U0126 treated cells with 400 μg/ml of MFX, (5) cells treated with 400 μg/ml of CFX alone, (6) U0126 treated cells with 400 μg/ml of CFX. **b** MFX and CFX augment apoptotic effects of cisplatin via ERK activation in pancreatic cancer cells. *(i)* Annexin-V assay of MIA PaCa-2 cells treated either alone with MFX and CFX (400 μg/ml) or in combination with cisplatin(CDDP) 20 μM for 48 h. *(ii)* Western blot analysis for pERK expression in MIA PaCa-2 cells treated either with MFX, CFX & CDDP alone or in combination for 48 h. Bar Graph represents the mean ± SEM of the fold change from three independent experiments. *p < 0.01, #p < 0.05 versus control

To confirm the role of ERK1/2 in FQ-induced apoptosis, we next did annexin assay in presence or absence of U0126. As shown in Fig. 8a, cells treated with U0126 for 1 h prior to addition of MFX/CFX (400 μg/ml) for 48 h, showed a significant reduction of percentage of apoptotic cells as compared to cells treated with MFX/CFX alone (p < 0.01). To check the role of p38 in CFX induced apoptosis, we did annexin assay in presence or absence of SB203580 (10 μM) along with CFX (400 μg/ml) for 48 h. Inhibition of p38 by SB203580 either in presence or absence of CFX did not showed significant change in apoptotic population, which confirms that FQ induced apoptosis is p38 independent (Additional file 4: Figure S4).

### Fluoroquinolones augments apoptotic effects of Cisplatin in pancreatic cancer cells via ERK activation

Cisplatin is very well known broad spectrum anticancer drug, which has been used in combination with other chemotherapeutic agents in advanced stages of pancreatic cancer [36]. Antiproliferative and apoptotic effects of Cisplatin have been attributed to activation of ERK in various cell lines [37]. Since, we also found that FQ used in our study show ERK dependent antiproliferative effect, we herein investigated if both the FQs could augment the apoptotic effects of cisplatin in pancreatic cancer cells. As shown in Fig. 8bi, MFX (400 μg/ml, p < 0.008) and CFX (400 μg/ml, p < 0.001) significantly enhances the apoptotic potential of Cisplatin (20 μM) when given in combination for 48 h. We also found the levels of pERK to be highly upregulated during combinatorial treatment compared to cells treated alone with FQ or cisplatin without changes in the levels of total-ERK (Fig. 8bii). Taken together, these results suggest that FQ augments the apoptotic effects of cisplatin via ERK activation.

## Discussion

Pancreatic carcinoma is the most aggressive forms of malignancy, that warrants more treatment options owing to its poor prognosis and single known drug therapy that to facing the challenge of resistance [38]. The present study characterizes the effects of MFX and CFX on cell cycle arrest and apoptosis signalling in pancreatic cancer cells. Herein we found that both the FQs caused cell growth inhibition, S-phase cycle arrest and apoptosis in pancreatic cancer cell lines MIA PaCa-2 and Panc-1 in a dose and time-dependent manner at physiologically relevant doses which are currently being used for the treatment of antibacterial infections in humans [39].

Literature reveals that coordinated action of Cyclin-A/Cyclin-E with their respective kinase (CDK-2) cause S-phase progression and inhibition of these cyclins and CDKs leads to accumulation of cells in S-phase [40]. As expected, in our current study too both the FQs significantly downregulated the levels of Cyclin-A, Cyclin-E, CDK2 without effecting the levels of G2-phase regulatory proteins cyclin-B1, pCDC2 and CDC25c. Our previous study [20] demonstrated that gatifloxacin caused S-phase arrest via TGFβ1-smad-p21 pathway in MIA PaCa-2 cells but herein we did not find any significant change in the levels of TGFβ1 after CFX treatment in both the cell lines and in fact significant decrease in the expression of TGFβ1 was observed after MFX treatment in Mia PaCa-2 cells (data not shown). Our results rule out the involvement of TGFβ1 in CFX and MFX induced S-phase arrest, and apoptosis. Our current findings were also in contrast to the study of Bourikas LA et al*.,* where they demonstrated that the anti-proliferative and immunoregulatory effect of CFX on human intestinal epithelial cells was mediated by TGFβ1 and it had no effect on Caco-2 a human colonic epithelial cell line that lacks functional TGFβ1 receptors [25]. The difference in mechanistic action of CFX in our study and their study could be attributed to the difference in origin of both the cell types. Increasing evidences in the literature show that different molecular pathways can be activated by diverse FQs in the same cell line [41].

Various evidences suggest that apoptosis is characterized by certain hallmarks such as phosphatidyl serine exposure to plasma membrane, activation of caspase −8, −9, −3 and DNA fragmentation [42]. Our annexin, cell cycle analysis, caspase activation, cleavage of poly(ADP-ribose) polymerase (PARP) and DNA fragmentation assay clearly demonstrates that both the FQs induces apoptosis in pancreatic cancer cell lines. We further observed CFX to be more potent than MFX in inhibiting proliferation and induction of apoptosis in both the cell lines. A decrease in full-length Bid, suggests a possible cross-talk between the intrinsic and extrinsic apoptotic pathway during FQ induced apoptosis in both the cell lines. Our study is in accordance to the reports by Aranha O et al., and Herold C et al., where they observed that CFX activates all the three caspases in colorectal carcinoma and bladder cancer cell lines at similar doses [14, 24].

There is mounting evidence implicating that members of the B-cell lymphoma-2 (BCL-2) family regulate the mitochondrial pathway of apoptosis by controlling the permeabilization of the outer mitochondrial membrane. The pro- and anti-apoptotic members such as Bax, Bak and Bcl-xL reside on outer mitochondrial membrane or cytosol and oligomerize under stress to facilitate the release of factors from mitochondria to trigger apoptosis. In the current study, MFX and CFX treatment resulted in significant increase in the expression of Bak along with decrease in Bax/Bcl2 ratio contributing towards the involvement of mitochondrial mediated intrinsic pathway in FQ mediated apoptosis. Modulation of anti-apoptotic and survival pathways is a strategy normally used to induce apoptosis in cancer cells. In our study we too observed that both the FQs not only downregulates anti-apoptotic proteins, upregulates pro-apoptotic proteins but also downregulates pro-survival proteins (c-Myc, AKT) in both the cell lines. AKT (Serine/Threonine kinases) is known to be involved in promoting cellular proliferation by regulating cell cycle and apoptosis [43]. Literature reveals that activated AKT not only prevents apoptosis but also confers resistance against chemotherapy and increasing evidences reveal that AKT inhibition prior to chemotherapy increases the efficacy of chemotherapeutic drugs [44].

Extracellular signal-related kinase (ERK) activation has been majorly known to regulate cellular proliferation and survival, but ERK1/2 pathway has also been known to be associated with various other processes such as differentiation, proliferation, transformation and apoptosis [35, 45, 46]. Several investigators independently reported activated ERK1/2 in induction of cell cycle arrest and apoptosis by various cytotoxic agents such as Asiatic acid, Pemetrexed, Phenethyl Isothiocyanate, Lauryl gallate, Taxol [47–51]. Literature also reveals that various anticancer agents such as etoposide, adriamycin and cisplatin also require prolonged activation of ERK1/2 as a prerequisite molecule for apoptosis induction in variety of primary or secondary immortalized and transformed cells [52]. Some studies suggest that ERK1/2 showed its apoptotic effects by targeting various downstream targets such as cMyc, Elk1 and p53 [53] whereas others suggest ERK1/2 mediated apoptosis is a result of balance between intensity and duration of pro- versus anti-apoptotic proteins [54]. Similar to our findings Cagnol et al., in their study reported that prolonged activation of ERK1/2 induces FADD independent caspase-8 activation and cell death [55]. In our study we found that activation of ERK1/2 is involved in FQ mediated apoptosis as suggested by the use of U0126 (a highly selective inhibitor of both MEK1 and MEK2, a type of MAPK/ERK kinase). Our results are in accordance to one of the recent report by Jemel-Oualha et al., where they have shown CFX to induce ERK mediated apoptosis in colon cancer cells [56]. In contrast to our study there is a report by Zheng et al., where ERK activation has been associated with gemcitabine resistance in pancreatic cancer cells [57]. However, the mechanism by which ERK1/2 activation mediates FQ-induced apoptosis varies depending on the context and needs further investigation.

Furthermore, in general tumour suppressor genes such as p53, p27 and p21 are up regulated during apoptosis but in our study they are down regulated. One should remember that tumour suppressor functions of genes/proteins are context-dependent and may be influenced by numerous factors, including cell type, the type of stress signal, microenvironment and their expression levels at the time of exposure to stress. Similar to our findings Tang et al., in their study reported that prolonged activation of ERK causes cell cycle arrest and apoptosis after DNA damage independent of p53 status [58]. How and why these tumour suppressor proteins are down regulated during FQ-mediated apoptosis remains an active area of investigation which is currently being investigated.

**Fig. 9:**
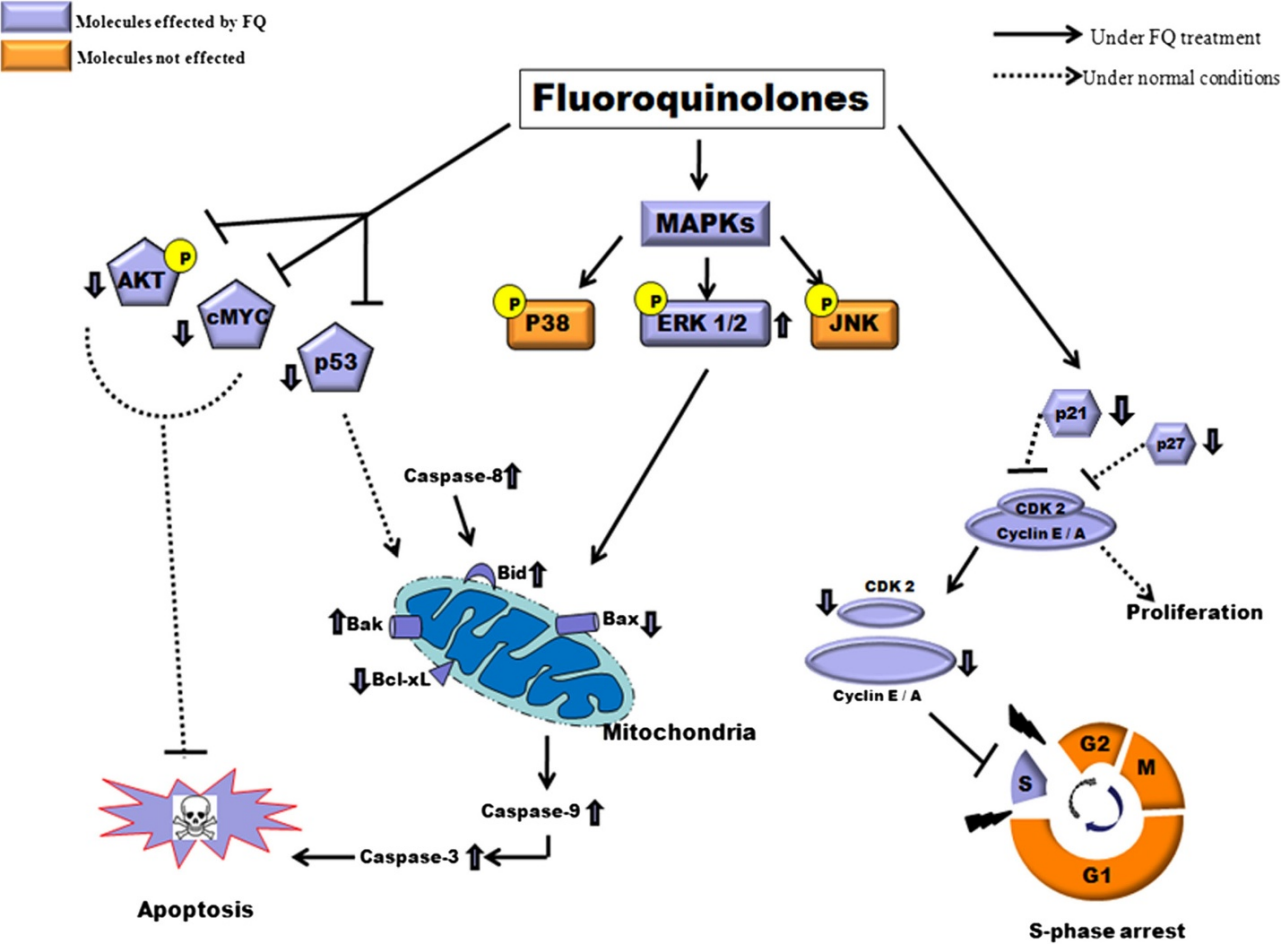
Proposed mechanism of action of MFX and CFX induced S-phase arrest and apoptosis. MFX and CFX causes S-phase arrest by decreasing the levels of Cyclin-A, Cyclin-E, CDK2, p21 and p27 in both the cell lines. Both FQs also leads to activation of extrinsic pathway of apoptosis via caspase-8 and ERK1/2 which then disrupts mitochondrial membrane potential via activation of Bid and proapoptotic Bak, as well as downregulates Bax and antiapoptotic protein Bcl-xL, which finally promotes activation of caspase-9,3 and leads to apoptosis. Furthermore MFX and CFX also suppresses cell survival pathway by downregulating the levels of pAKT and cMYC

According to above results we herein propose a model for mode of action of both the FQs in pancreatic cancer cells as shown in Fig. 9.

## Conclusion

We demonstrated that induction of apoptotic cell death and S-phase arrest contributes to the anti proliferative effect of MFX and CFX in pancreatic cancer cell lines, MIA PaCa-2 and Panc-1 cells. CFX was found to be more potent in inducing apoptosis than MFX in both the cell lines. In addition we showed that MFX and CFX not only cause S-phase arrest and apoptosis individually, but also augments Cisplatin induced apoptosis in human pancreatic cancer cells in ERK1/2 dependent manner. We believe that our data would contribute to the development of MFX and CFX as potential neo-adjuvant chemotherapeutic agents for the treatment of pancreatic cancer. However, one major limitation of the study is that all data are derived from *in vitro* systems and *in vivo* validation is extremely important for these agents to become as therapeutics for cancer.

## Additional files

Additional file 1: Figure S1. MFX and CFX induced apoptosis is caspase-dependent in both the cell lines. Western blot of cleaved caspase-8, 9, 3, and PARP under the effect of MFX (400 μg/ml) and CFX (400 μg/ml) in presence or absence of zVAD-fmk (Pan caspase inhibitor, 20 μM) (JPEG 377 kb)
Additional file 2: Figure S2. MFX and CFX induced apoptosis is independent of p53 status. (i) Annexin-V assay ofHCT116 p53+/+ and p53 −/− treated with MFX and CFX (400 μg/ml) for 24 h. Bar graph represents mean ± SEM from three independent experiments, where vertical axis represents % apoptotic cells and horizontal axis represents MFX and CFX (400 μg/ml)concentration. (ii) Western blot analysis for p53 expression in HCT116 p53+/+ and p53 −/− cell lines treated with MFX and CFX in a dose dependent manner **(**0**–**400 μg/ml**)** for 24 h. (JPEG 335 kb)
Additional file 3: Figure S3. MFX and CFX do not affect G2-phase associated regulatory proteins. Western blot analysis of G2-phase regulatory Cyclins and CDKs in MIA PaCa-2 and Panc-1 cells treated with MFX and CFX in a dose dependent manner. GAPDH was used as loading control. (JPEG 397 kb)
Additional file 4: Figure S4. CFX induced apoptosis is independent of p38 in pancreatic cancer cells. (i) Annexin V-PE assay in MIA PaCa-2 cells treated with CFX in presence and absence of SB203580 (10 μM). Results are represented in the form of bar graph where vertical axis represents % apoptotic cells and horizontal axis represents presence or absence of CFX and SB203580. Bar graph represents mean ± SEM from three independent experiments. (ii) Western blot analysis for the knockdown efficiency of p38 inhibitor (SB203580) in presence and absence of CFX. (JPEG 278 kb)

## Abbreviations

FQ: Fluoroquinolone
MFX: Moxifloxacin
CFX: Ciprofloxacin
CDDP: Cisplatin
ERK: Extracellular-signal-regulated kinase
JNK: c-JUN N-terminal kinase
CDK: Cyclin dependent kinase
MAPK: Mitogen-activated protein kinase
PARP: Poly(ADP-ribose) polymerase
U0126: ERK inhibitor
EDTA: Ethylenediaminetetraacetic acid
TGFβ1: Transforming growth factor- β1
SB203580: p38 inhibitor
MTT: 3-(4,5-dimethylthiazol-2-yl)-2,5-diphenyltetrazolium bromide
