# Supplementary figures and images for: Moxifloxacin and ciprofloxacin induces S-phase arrest and augments apoptotic effects of cisplatin in human pancreatic cancer cells via ERK activation
Source: BMC Cancer. 2015 Aug 11;15:581. doi: 10.1186/s12885-015-1560-y (PMC4531397; doi:10.1186/s12885-015-1560-y)

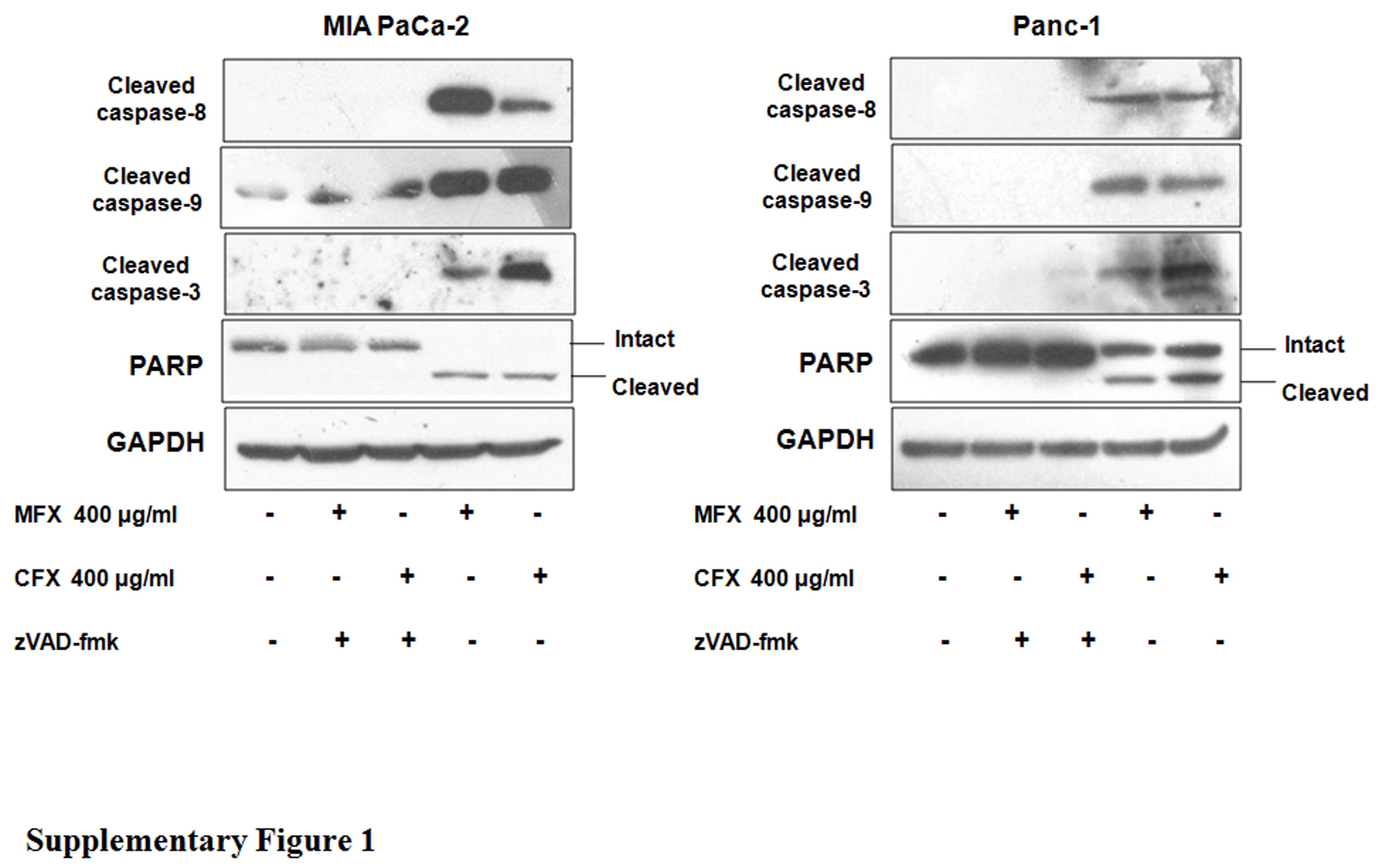

Supplement: Additional file 1: Figure S1. — MFX and CFX induced apoptosis is caspase-dependent in both the cell lines. Western blot of cleaved caspase-8, 9, 3, and PARP under the effect of MFX (400 μg/ml) and CFX (400 μg/ml) in presence or absence of zVAD-fmk (Pan caspase inhibitor, 20 μM) (JPEG 377 kb) [file 12885_2015_1560_MOESM1_ESM.jpeg]

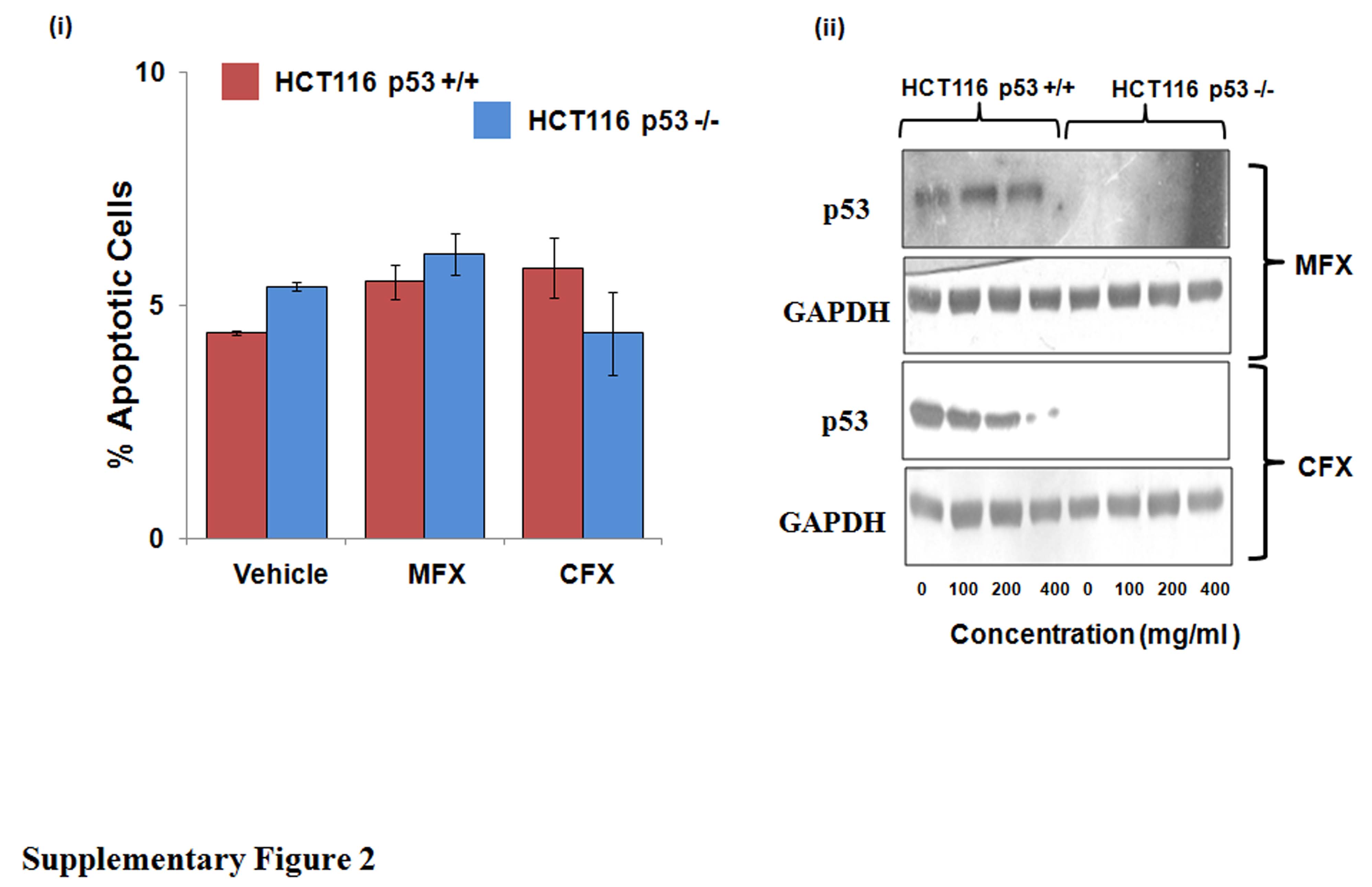

Supplement: Additional file 2: Figure S2. — MFX and CFX induced apoptosis is independent of p53 status. (i) Annexin-V assay ofHCT116 p53+/+ and p53 −/− treated with MFX and CFX (400 μg/ml) for 24 h. Bar graph represents mean ± SEM from three independent experiments, where vertical axis represents % apoptotic cells and horizontal axis represents MFX and CFX (400 μg/ml)concentration. (ii) Western blot analysis for p53 expression in HCT116 p53+/+ and p53 −/− cell lines treated with MFX and CFX in a dose dependent manner (0–400 μg/ml) for 24 h. (JPEG 335 kb) [file 12885_2015_1560_MOESM2_ESM.jpeg]

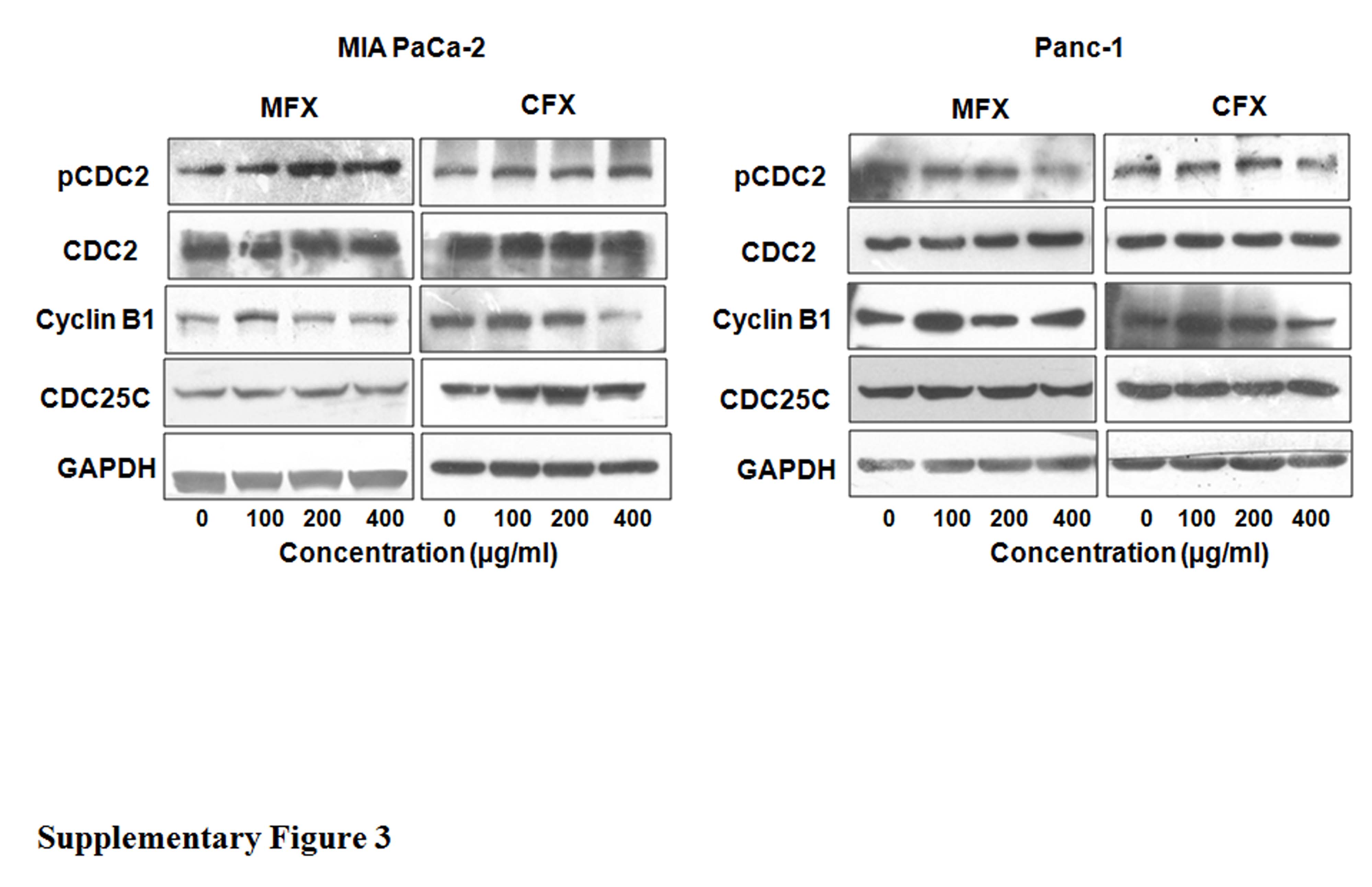

Supplement: Additional file 3: Figure S3. — MFX and CFX do not affect G2-phase associated regulatory proteins. Western blot analysis of G2-phase regulatory Cyclins and CDKs in MIA PaCa-2 and Panc-1 cells treated with MFX and CFX in a dose dependent manner. GAPDH was used as loading control. (JPEG 397 kb) [file 12885_2015_1560_MOESM3_ESM.jpeg]

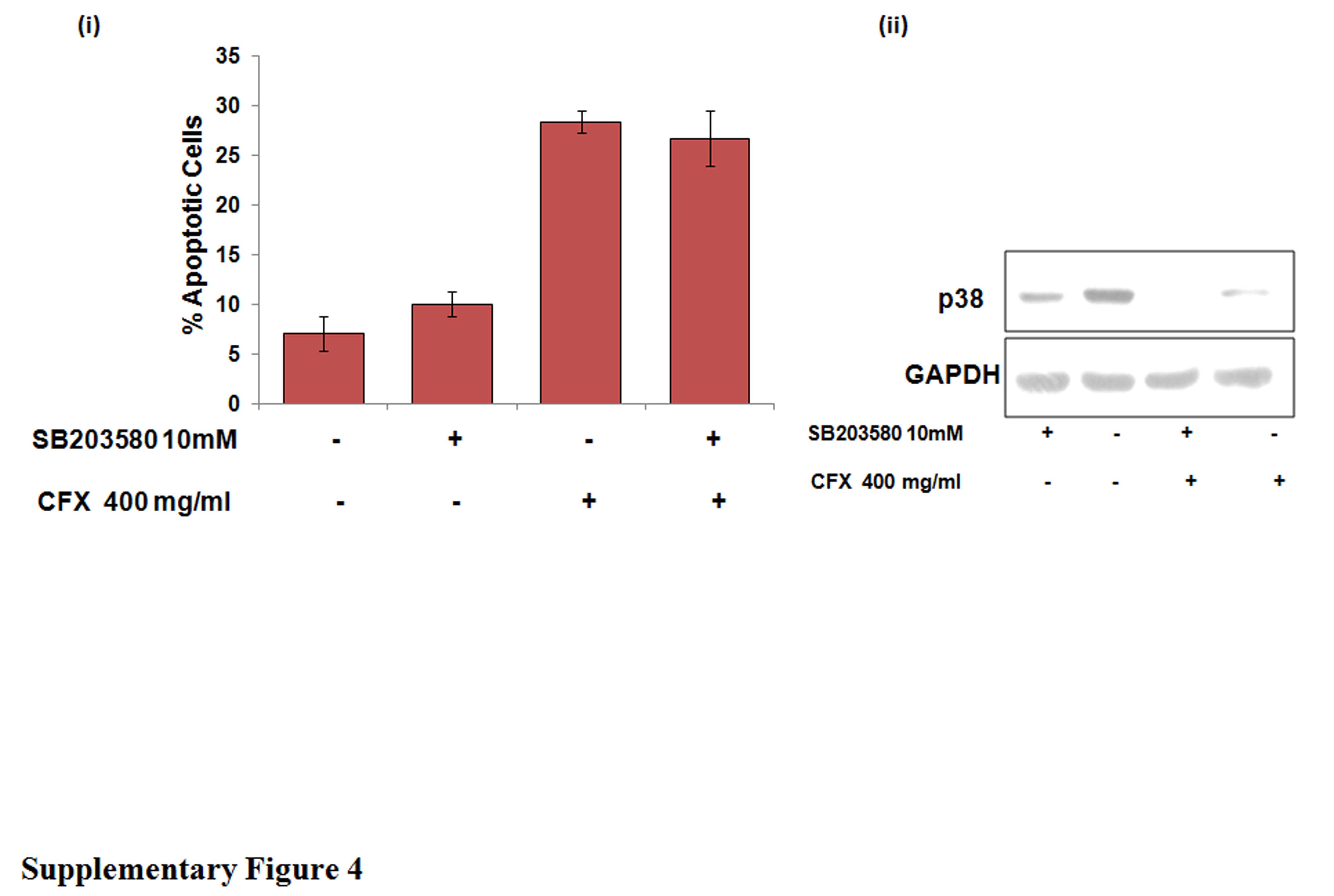

Supplement: Additional file 4: Figure S4. — CFX induced apoptosis is independent of p38 in pancreatic cancer cells. (i) Annexin V-PE assay in MIA PaCa-2 cells treated with CFX in presence and absence of SB203580 (10 μM). Results are represented in the form of bar graph where vertical axis represents % apoptotic cells and horizontal axis represents presence or absence of CFX and SB203580. Bar graph represents mean ± SEM from three independent experiments. (ii) Western blot analysis for the knockdown efficiency of p38 inhibitor (SB203580) in presence and absence of CFX. (JPEG 278 kb) [file 12885_2015_1560_MOESM4_ESM.jpeg]
